# The spaceflight contrast sensitivity hypothesis and its role to investigate the pathophysiology of spaceflight-associated neuro-ocular syndrome

**DOI:** 10.3389/fopht.2023.1229748

**Published:** 2023-09-05

**Authors:** Ethan Waisberg, Joshua Ong, Nasif Zaman, Phani Paladugu, Sharif Amit Kamran, Alireza Tavakkoli, Andrew G. Lee

**Affiliations:** ^1^ Department of Ophthalmology, University of Cambridge, Cambridge, United Kingdom; ^2^ Moorfields Eye Hospital, NHS Foundation Trust, London, United Kingdom; ^3^ Michigan Medicine, University of Michigan, Ann Arbor, MI, United States; ^4^ Human-Machine Perception Laboratory, Department of Computer Science and Engineering, University of Nevada, Reno, NV, United States; ^5^ Brigham and Women’s Hospital, Harvard Medical School, Boston, MA, United States; ^6^ Center for Space Medicine, Baylor College of Medicine, Houston, TX, United States; ^7^ Department of Ophthalmology, Blanton Eye Institute, Houston Methodist Hospital, Houston, TX, United States; ^8^ The Houston Methodist Research Institute, Houston Methodist Hospital, Houston, TX, United States; ^9^ Departments of Ophthalmology, Neurology, and Neurosurgery, Weill Cornell Medicine, New York, NY, United States; ^10^ Department of Ophthalmology, University of Texas Medical Branch, Galveston, TX, United States; ^11^ Department of Ophthalmology, University of Texas MD Anderson Cancer Center, Houston, TX, United States; ^12^ Department of Ophthalmology, Texas A&M College of Medicine, Bryant, TX, United States; ^13^ Department of Ophthalmology, The University of Iowa Hospitals and Clinics, Iowa, IA, United States

**Keywords:** spaceflight-associated neuro-ocular syndrome, SANS, contrast sensitivity, head-mounted technology, virtual reality

## Introduction

Currently, on the International Space Station (ISS) scheduled astronaut vision assessments include a laptop-based static visual acuity test, a self-reported survey, and an Amsler grid test (1).

However, these high-contrast visual tests may not be sensitive enough to capture neuro-ophthalmic disease or disease of the optic nerve. Contrast sensitivity (CS) is a test currently performed when an astronaut is clinically indicated during a spaceflight. We believe that regular CS assessments may be needed to better understand spaceflight-associated neuro-ocular syndrome (SANS).

SANS is a collection of pathologic and physiologic findings seen in astronauts after long-duration spaceflight (LDSF) (2). SANS has been observed to affect astronaut vision and key ocular structures, which are critical considerations for even longer missions in microgravity such as the mission to Mars (3). The pathophysiology of SANS remains poorly understood; however, having a further understanding of terrestrial analogs of SANS, such as idiopathic intracranial hypertension (IIH), may play a key role in better understanding this condition. SANS and terrestrial IIH share similarities such as optic disc edema and globe flattening. Retinal and choroidal folds have also been reported in both SANS and IIH. Although there is not a perfect terrestrial analog for SANS, this hypothesis and the clinical similarities suggest that IIH may serve as a close analog for SANS. However, several differences exist between SANS and IIH. Astronauts have not reported diplopia, pulse-synchronous tinnitus, or severe headache during LDSF. IIH is a metabolic disease driving CSF hypersecretion, whereas SANS is directly related to the microgravity environment. There is also a much larger proportion of individuals with SANS with asymmetric or unilateral optic disc edema than with the typical symmetrical optic disc edema in IIH (4). Post-flight lumbar puncture (LP) opening of pressures in the astronauts with SANS has also shown normal to slightly elevated values, which differs from IIH (2). However, it is important to note that LPs have been done, in some cases, weeks, months, and years following LDSF. In addition, parabolic flight studies have shown that there are no increases in intracranial pressure (ICP) that are above supine level (5). It should be noted that parabolic flights allow for only minutes of weightlessness in contrast to LDSF, which last several months. The name “SANS” reflects other potential etiologies for these findings, including the ocular glymphatic system, upward brain shift, cerebral blood volume pulsatility, and genetic/metabolic factors (4). Anemia has recently been implicated in the terrestrial development of pseudotumor cerebri (6–9). Astronauts have a lower red blood cell mass than their preflight values, but these do not meet a clinical definition of anemia, so whether or not this has any role in SANS pathogenesis is unknown.

Rehman et al. recently reported changes in CS in patients with IIH (10). This study had 10 IIH patients undergo the Spaeth–Richman Contrast Sensitivity (SPARCS) test, an online test that evaluates contrast sensitivity in one central quadrant and four peripheral quadrants. This novel study demonstrated that peripheral CS (in the inferonasal, inferotemporal, and superonasal quadrants) was significantly more affected than central CS in IIH patients, indicating that peripheral CS testing may play a role in the clinical evaluation of IIH. These results, however, may also have extraterrestrial implications for CS testing in SANS. This syndrome is one of the largest potential barriers to future crewed spaceflight and the potential role of CS in SANS continues to expand. Maintaining adequate CS is vital for mission performance to allow astronauts to discriminate finer details or objects from their background. We also hypothesize that CS likely decreases during microgravity due to long-term SANS. The prolonged periods of optic disc edema seen in SANS may lead to retinal ganglion damage at the optic nerve head. As also mentioned in a separate study by Rehman et al., the parvocellular pathway and magnocellular pathway may be affected retinal ganglion cell damage during optic disc edema (11). This hypothesis that CS decreases during LDSF is much more nuanced and may involve these different pathways. The magnocellular pathway has a higher sensitivity for higher temporal, lower spatial frequencies, which enables contrast detection over a broad luminance range, whereas the parvocellular pathway has a higher sensitivity for lower temporal, higher spatial frequencies, which helps with chromatic processing (11). By further understanding how these different pathways are affected during spaceflight, we can better characterize how SANS affects the neuro-ophthalmic system.

## Discussion

**Figure 1:**
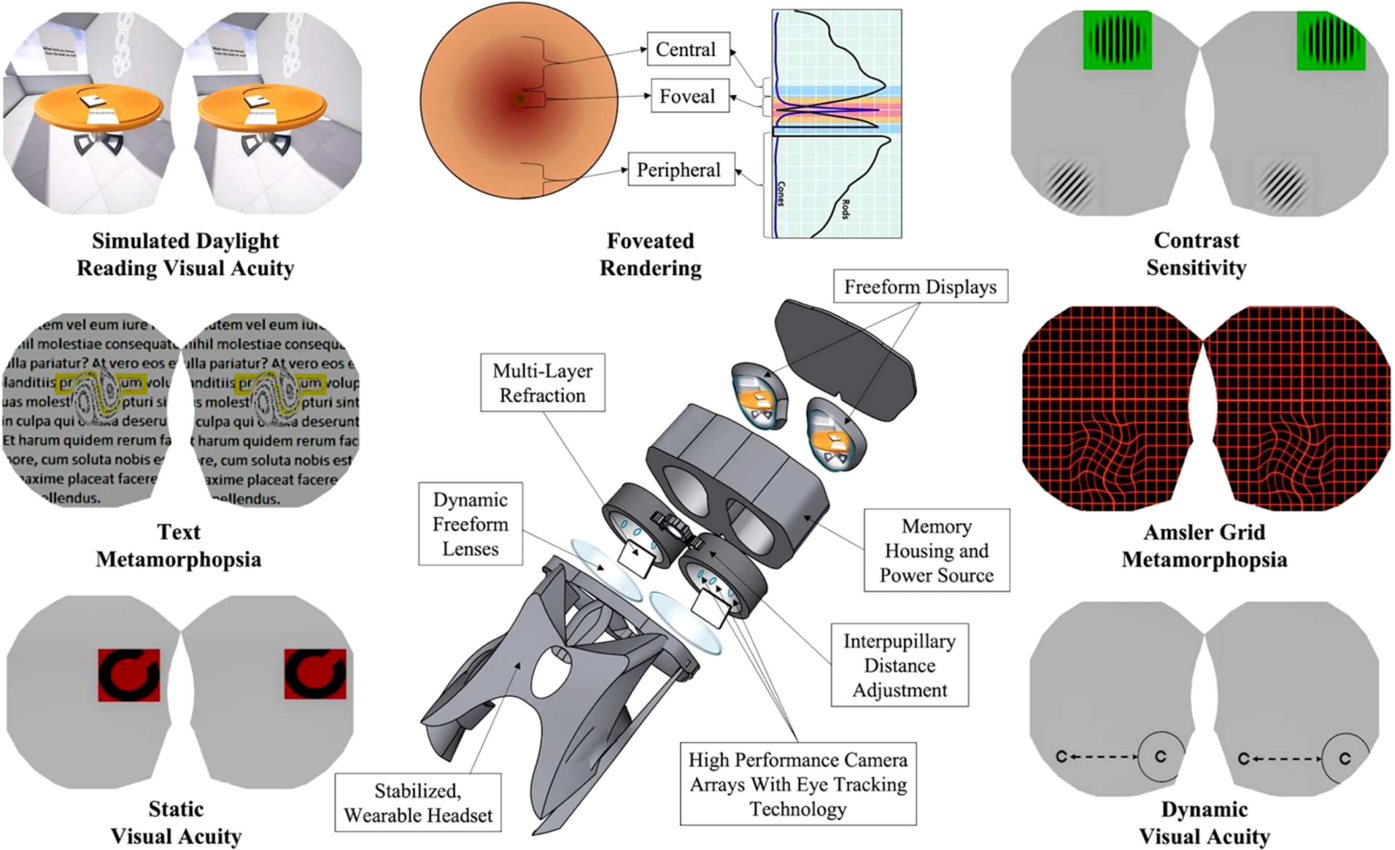
Diagram of the head-mounted visual assessment system. Reprinted with permission from Ong, J., Tavakkoli, A., Zaman, N. et al. Terrestrial health applications of visual assessment technology and machine learning in spaceflight associated neuro-ocular syndrome. *NPJ Microgravit*y 8, 37 (2022). https://doi.org/10.1038/s41526-022-00222-7. Creative Commons Attribution 4.0 International License.

The novel findings from Rehman et al. strongly support the fact that CS testing may be another potential assessment opportunity in SANS. Laptop-based CS testing is currently available onboard the ISS. However, CS is not a routine test, but is performed only when astronauts are clinically indicated. Laptop-based testing also requires additional time to set up and stow, which is a potential rate-limiting step for astronauts on the ISS. To provide more efficient visual assessment testing during LDSF to monitor vision and SANS, our group is currently developing a head-mounted, multimodal visual assessment system, including CS tests (**Figure 1**). This work is supported by the National Aeronautics and Space Administration (NASA) and is part of our development of a non-invasive framework for astronaut vision, with the goal of monitoring subtle vision changes that occur during spaceflight (12, 13). The proposed assessment platform seeks to provide rapid, lightweight, convenient, easy-to-use, and portable testing. Head-mounted technology reduces variability from external illumination, offers eye-tracking technology, and can be self-operated by the individual astronaut (14).

Although the pressure changes in the head when going from supine to head-down tilt differ profoundly from the changes produced when going from supine to weightlessness, strict head-down-tilt bed rest has reproduced some elements of SANS (e.g., optic disc edema and chorioretinal folds) (15). As such, testing such technology for future spaceflight with head-down-tilt bed rest is a future direction prior to onboard deployment (16). The self-operated accessibility and lightweight aspects of head-mounted technology have clear advantages over other traditional and more bulky visual assessment methods, especially for head-down-tilt bed-rest subjects. The head-mounted device (including CS assessment) may also find future terrestrial applications to allow for more convenient, rapid, and frequent visual assessments, as well as for opportunities for at-home testing (17). Further research to establish test–retest reliability will also be essential when bringing this technology into the clinical setting (18).

The novel reporting on the utility of CS for IIH supports the consideration of CS assessment for SANS. These advances in knowledge may help to produce more effective countermeasures against SANS to protect astronaut vision for future LDSF missions, including the planned future crewed mission to Mars.

## Author contributions

EW—design and writing. JO—design and writing. NZ—review and intellectual support. PP—review and intellectual support. SK—review and intellectual support. AT—review and intellectual support. AL—review and intellectual support. All authors contributed to the article and approved the submitted version.
